# Fructose: A Dietary Sugar in Crosstalk with Microbiota Contributing to the Development and Progression of Non-Alcoholic Liver Disease

**DOI:** 10.3389/fimmu.2017.01159

**Published:** 2017-09-19

**Authors:** Jessica Lambertz, Sabine Weiskirchen, Silvano Landert, Ralf Weiskirchen

**Affiliations:** ^1^Institute of Molecular Pathobiochemistry, Experimental Gene Therapy and Clinical Chemistry (IFMPEGKC), RWTH University Hospital Aachen, Aachen, Germany; ^2^Culture Collection of Switzerland AG (CCOS), Wädenswil, Switzerland

**Keywords:** fructose, gut-liver-axis, inulin, insulin resistance, microbiota, SCFA, probiotics, prebiotics

## Abstract

Fructose is one of the key dietary catalysts in the development of non-alcoholic fatty liver disease (NAFLD). NAFLD comprises a complex disease spectrum, including steatosis (fatty liver), non-alcoholic steatohepatitis, hepatocyte injury, inflammation, and fibrosis. It is also the hepatic manifestation of the metabolic syndrome, which covers abdominal obesity, insulin resistance, dyslipidemia, glucose intolerance, or type 2 diabetes mellitus. Commensal bacteria modulate the host immune system, protect against exogenous pathogens, and are gatekeepers in intestinal barrier function and maturation. Dysbalanced intestinal microbiota composition influences a variety of NAFLD-associated clinical conditions. Conversely, nutritional supplementation with probiotics and preobiotics impacting composition of gut microbiota can improve the outcome of NAFLD. In crosstalk with the host immune system, the gut microbiota is able to modulate inflammation, insulin resistance, and intestinal permeability. Moreover, the composition of microbiota of an individual is a kind of fingerprint highly influenced by diet. In addition, not only the microbiota itself but also its metabolites influence the metabolism and host immune system. The gut microbiota can produce vitamins and a variety of nutrients including short-chain fatty acids. Holding a healthy balance of the microbiota is therefore highly important. In the present review, we discuss the impact of long-term intake of fructose on the composition of the intestinal microbiota and its biological consequences in regard to liver homeostasis and disease. In particular, we will refer about fructose-induced alterations of the tight junction proteins affecting the gut permeability, leading to the translocation of bacteria and bacterial endotoxins into the blood circulation.

## Diet Influences Microbiota

Diet and connected nutritional status depict important, modifiable factors of human health. Major determinants are gut microbial community (microbiota) and its genes (microbiome) (1, 2). In turn, the gastrointestinal microbiota is highly influenced by the diet of the host. Turnbaugh et al. showed that a shift from a Mediterranean diet (MD) rich in polysaccharides to a Western diet, high in fat and sugar, low in fiber, is able to alter microbiota within a day (1). Similarly, diets high in sugar significantly decrease the microbial diversity in the gut after just 1 week (3).

Also another more recent study has shown that the consuming of a MD promotes a gut flora enriched in polysaccharide-degrading microbes and end products of polysaccharide fermentation, whereas in contrast, a Western diet leads to a community of microorganisms in the digestive tract that mostly contain proteolytic microbes and end products of protein and fat metabolism (4).

During metabolism, a part of the ingested food such as dietary fiber or resistant starch escapes digestion in the small intestine, reaches the colon and is fermented by gut microbes, which produce products such as short-chain fatty acids (SCFA), trimethylamine, ammonia, and hydrogen sulfide, which have beneficial impact on its host. With the diet also the economy of microbiota changes, because the different strains favor different environment and nutrients. Differences in microbial structure and function are reflected in the diversity of intestinal metabolites. For example, SCFA such as acetate, butyrate and propionate representing end products of fermentation of complex carbohydrates, were significant higher in samples taken from Egyptian teenagers consuming a MD than in teenagers from the United States absorbing a typical Western diet (4). SCFA are known to inhibit inflammation and obesity (5), while the end products resulting from lipid and protein degradation are associated with arteriosclerosis and colon cancer (6). Moreover, intestines of teenagers consuming a Western diet have elevated amino acid content and higher levels of lipid metabolism-associated compounds, and higher concentrations of 1-methylhistamine indicating allergic reactions and suggesting that the intestinal microbiota is majorly determined by the host diet (4). In line with these findings, the Western diet was found to be one of the causes for metabolic diseases such as non-alcoholic fatty liver disease (NAFLD). In combination with stress exposure, diets enriched in fat and fructose are even able to modulate brain immunity, and increase metabolic vulnerability to conditions associated with NAFLD, arteriosclerosis and cardiovascular disease (7). In contrast, the consumption of components of the MD enriched in olive oil, fish, nuts, whole grains, fruits, and vegetables is negatively correlated with the pathogenesis of NAFLD (8).

## Fructose in Human Diets

Fructose is an integral part of human diets. This monosaccharide appears naturally in ripe fruits, honey, and in small amounts in vegetables including carrot, onion, sweet potato, and paprika. In the 1960s, fructose has been shown to have positive effects in the treatment of diabetes because it does not need insulin to be metabolized. Fructose feeding had no influence on fasting blood glucose and its excretion into the urine (9, 10). This sugar was described as a “useful therapeutic agent” stabilizing blood glucose in diabetic patients to the normal fasting level, improving the overproduction of acetone, restoring the nitrogen balance, and decreasing the loss of water (9). All these changes were induced without any changes in insulin therapy, just by substitution of fructose for glucose (9). Similarly, intravenous administration of fructose was effective in the treatment of diabetic ketosis (11). In the 1960, high fructose corn syrup was inserted in the food industry as a substitute of sugar and the intake increased, while the clinical importance of this sugar is still discussed controversially and in the focus of research (12). In particular, fructose was identified as a sugar affecting lipid metabolism by rising plasma triglycerides and fasting plasma free fatty acids. Therefore, the usage of this sugar was challenged for treatment of diabetes (10). Today, the view on fructose has changed dramatically. It is now handled as a risk factor in the development of obesity and several metabolic disturbances. In addition, fructose worsens symptoms in irritable bowel syndrome (IBS), an inflammatory condition characterized by abdominal pain, diarrhea, and bloating (13). Human studies performed in patients suffering from IBS showed a high prevalence of fructose malabsorption up to 64% (14). Interestingly, also patients tested negative for disorders in fructose metabolism showed abdominal symptoms after fructose ingestion suggesting fructose intolerance as a highly common condition (13). In both studies, 25 g of fructose were orally administered in a fructose breath test to prove malabsorption. Noteworthy, beverages usually contain 10 g sugar/100 mL. On the other side, if beverages are sweetened with high-fructose corn syrup such as HFCS-55 containing 55% of fructose, consumers absorb 27.5 g fructose drinking a volume of 500 mL. This amount is more than tested in the mentioned human studies and again emphasizes the fact that fructose in beverages and food nutrients should be critically considered as risk factors for inflammatory diseases.

## Microbiota Impacts the Host Immune System

Bacteria in the gut are responsible for digestion and producing essential vitamins and minerals (2). In addition, they are important for host physiology, digestion of indigestible food materials, and production of bile acids (15). Moreover, the gut microbiota has an influence on several immune functions, protects against pathogens, and joins in the maturation of the gut barrier (16).

Our intestine is an individual immunological site where interaction between microbiota and its host takes place (17). If the homeostasis between microbiota and host is disturbed, inflammation and cancer can occur (17). The crosstalk of the intestinal microbiota with the immune system of the host can modulate insulin resistance and intestinal permeability (16). Furthermore, it has an impact on the body weight. This could be demonstrated by transplant experiments in which gut communities isolated from obese mice fed a Western diet were transplanted into germ-free recipient mice. The colonization produced adiposity in the recipient mice within 2 weeks (1).

## Short-Chain Fatty Acids, Metabolites of Microbiota Affect the Immune System and Influence Disease Progression

Not only the microbiota itself but also its products influence the metabolism, energy intake and immunity (18). The human digestive system is restricted of debranching enzymes necessary to digest fibers and higher non-digestible carbohydrates such as pectin, inulin, gums, and cellulose (15). When these nutrients reach the distal gut, they stimulate growth and activity of bacteria that can ferment these compounds (19). During this microbial fermentation, SCFAs are formed influencing gut health and affecting as signaling molecules metabolism and function of peripheral tissues (20). There are three main SCFA, namely acetate, propionate and butyrate, which are the most important gut-derived products, acting as signal transduction molecules with epigenetic impact (21). Almost 10% of the human energy requirement per day is provided by SCFA *via* the host colonic epithelial cells (22). In particular, butyrate is the main energy source for normal colonic epithelial cells, protecting against colorectal, cancer, and inflammation (23). Especially, the fermentation of butyrate is highly inducible by lactate through lactate-utilizing bacterial strains converting starch and fructooligosaccharides (FOS) into butyrate (24, 25). In line, high intake of non-digestible fibers caused an accumulation of lactate exhibiting a low pKa value and provoking metabolic acidosis (25). Therefore, the utilization of lactate by lactate-utilizing microbiota is important in the context of SCFAs formation. SCFAs are absorbed by both passive diffusion and *via* monocarboxylic acid transporters (26). However, the production and molecular effects of SCFAs is presently controversially discussed. On one hand, SCFAs provide energy for the body (17, 21), while on the other site they can lead to extra fat deposition in the body and obesity (27). Moreover, SCFAs can directly influence several different functions such as satiety and host metabolism, improve glucose homeostasis, and insulin sensitivity (20). Although SCFAs are a rich source of calories, their intestinal production is associated with lean body weight, reduced inflammation, and increased satiety (28). They are ligands for receptors regulating appetite, inhibiting gastric emptying, while at the same time stimulating insulin secretion. Thereby SCFAs influence eating habits and the metabolism of the host and prevent exaggerate energy intake and obesity (15). Moreover, butyrate is an important molecule in the lipid metabolism of the host used to synthesize cholesterol and palmitate, while propionate is the principal gluconeogenic substrate decreasing hepatic glucose production (22, 29). Furthermore, butyrate has direct anti-inflammatory potential in the gut and the brain that helps to maintain the gut-barrier integrity and protects against the influx of toxins (29, 30).

Acetate promotes antilipolytic activity and it may also have metabolically beneficial effects in white adipose tissue (17). Furthermore, it mediates effects in the central nervous system suppressing appetite (31). So it is obviously that SCFAs affect metabolism and energy homeostasis by impacting glucose homeostasis, insulin sensitivity, skeletal muscle and liver tissue functions, adipose tissue biology (20, 21). With regard to the current literature, there is no reported direct interaction between the monosaccharide fructose and SCFA. Fructose consumption influences the microbiota and therefore affects the composition of SCFA in the gut. Interestingly, cross-feeding studies between *Bifidobacterium* and two acetate-converting, butyrate-producing strains (i.e., *Anaerostipes caccae* DSM 14662 and *Roseburia intestinalis* DSM 14610) in which fructose was used as the sole energy source allowed oligofructose breakdown by the strains not able to degrade the substrate itself (32). In this coculture study, *Bifidobacterium* releases small amounts of free fructose and acetate during degradation of fructooligosacharides.

Noteworthy, all three SCFA were able to decrease tight junction permeability, protecting barrier properties during increased microvascular leakage, which is the reason for several disease conditions (33). However, the effective concentration of each SCFA necessary to mediate these effects is different. The most effective concentration of butyrate (0.5 mmol/L) to decrease paracellular permeability was lower than that of propionate (1 mmol/L) and acetate (32 mmol/L) suggesting that in particular butyrate producers in the gut strengthens the gut barrier (33). SCFAs can influence the enteric nerve system, stimulating motility and secretion activity or affecting immune cells thereby reducing inflammation and tumorigenesis (34). Similar to the effect of histone deacetylases, SCFAs have anti-inflammatory and immune-suppressive functions and act as modulators in immune homeostasis and cancerogenesis (17, 21).

Short-chain fatty acids are able to reduce the pH of the gut, altering the composition of microbiota. Changing the pH from 5.5 to 6.7 favors the population of gut microbiota that produces propionate, while reducing the pH to 5.5 favored bacteria producing butyrate (35). This process maintains the gut homeostasis and economy. As outlined above, butyrate is already more effective at lower concentrations than propionate and acetate, and it need to be considered that butyrate at higher concentrations may provide too much energy which can promote obesity.

Dietary fiber is known to promote weight loss and improve glycemic control. High fat diet enriched in SCFAs protected from obesity and improved glucose tolerance (36). However, studies on propionate have shown that the effects of SCFA are tissue specific. While propionate-dependent gluconeogenesis had a beneficial effect on metabolic health in the small intestine, it was detrimental in the liver (17).

The gut microbiota also produces diet-dependent many other metabolites such as secondary bile acids and amino acid derivatives that have essential functions in the body. An increased production of intestinal bile acid occurs in a high fat diet. Bile plays important roles in lipid and carbohydrate metabolism and also in mediating inflammatory responses (37). Diets rich in saturated fatty acids can change the composition of the bile acids and promote dysbiosis. The changed microbiota is then able to inhibit hepatic gluconeogenesis and glycolysis and impact insulin sensitivity (16). As a consequence, bile-sensitive bacteria, such as *Prevotella* will be less prevalent, and more bile-tolerant bacteria will be predominant. In contrast to these findings, the bile acids chenodeoxycholic acid and cholic acid have been shown to improve the conditions of fructose-induced NAFLD by regulating intestinal transepithelial permeability and prohibiting the translocation of bacterial endotoxin from the gut into the portal plasma, thereby diminishing the activation of hepatic Kupffer cells (38). Additionally, these bile acids protected against the loss of tight junctions proteins in the intestinal epithelium. *Vice versa* the composition of bile acids is mutual influenced by diet and microbiota modulating the immune system of the host and impacting the development of diseases such as NAFLD.

## How Does Fructose Contribute to NAFLD and Non-Alcoholic Steatohepatitis (NASH)

Non-alcoholic fatty liver disease is one of the most frequent hepatic disease (39). It incorporates a disease spectrum, which includes steatosis (fatty liver), NASH, and cirrhosis. While simple steatosis is reversible, NASH provokes hepatocyte injury, inflammation, and fibrosis that can aggravate to cirrhosis, liver failure, and even hepatocellular carcinoma (40). Moreover, NAFLD is associated with characteristics of the metabolic syndrome, which includes abdominal obesity, insulin resistance, glucose intolerance or type 2 diabetes mellitus, and dyslipidemia syndrome (41–43).

Gut microbiota has been recognized as the main environmental factor promoting metabolic diseases (28). Multiple studies reported fructose as a critical factor contributing to NAFLD progression by modulating intestinal microbiota (Table 1). It performs crosstalk with its host, maintaining the host’s energy homeostasis and stimulating the host’s immunity. Shifts in this composition can result in alterations of the symbiotic relationship, which can promote metabolic diseases (28). The key bacterial products involved in the pathogenesis of NAFLD are lipopolysaccharides (LPS) (44). LPS is derived from gram-negative bacteria and are known to critically contribute to inflammation-related processes and insulin resistance. It is able to cross the gastrointestinal mucosa *via* leaky tight junctions or infiltrating chylomicrons (28). Recent studies connected NAFLD to disturbances in the gut microbial environment. The microbial composition differed between healthy individuals and NAFLD patients (45). In line, a diet enriched in fructose not only induced NAFLD but also negatively affected the gut barrier and the microbiota, leading to dysbiosis (46).

**Table 1:** Fructose in the crosstalk with microbiota in the pathogenesis of NAFLD.

Species	Treatment	Findings	Conclusion	Reference
Mouse	Diet high in saturated fat, fructose, and cholesterol for 8 weeks	*F11r*^−/−^ mice with defects in intestinal epithelial permeability developed more severe steatohepatitis than control mice.	Diet-induced microbial dysbiosis contribute to the development of NASH.	Rahman et al. (47)
	High fat diet with 10% fructose	Addition of *Lactobacillus paracasei* reduced expression of inflammatory markers (*Tlr4, Nox-4, Tnf-*α, *MCP-1, IL-4*) and number of Kupffer cells, and induced M2-dominant Kupffer cell polarization.	*Lactobacillus paracasei* attenuates hepatic steatosis with M2-dominant Kupffer cell polarization.	Sohn et al. (48)
	30% fructose in drinking water	Endotoxin levels in portal blood and lipid peroxidation as well as TNF-α expression were significantly increased in fructose-fed mice. Hepatic lipid accumulation was lowered by concomitant treatment with antibiotics.	Fructose increase intestinal translocation of endotoxin leading to liver damage.	Bergheim et al. (49)
	30% fructose in drinking water for 8 weeks	In fructose-fed *tlr4* mice, hepatic triglyceride accumulation was significantly reduced by approximately 40% in comparison to fructose-fed wild type mice.	Fructose-induced NAFLD is associated with intestinal bacterial overgrowth and increased intestinal permeability.	Spruss and Bergheim (50)
	Chronic consumption of 30% fructose solution with or without *Lactobacillus casei Shirota*	Treatment with *Lactobacillus casei Shirota* attenuated activation of TLR4 signaling.	Treatment with *Lactobacillus casei Shirota* protects against the onset of fructose-induced NAFLD.	Wagnerberger et al. (51)
	30% fructose solution for 8 weeks	Occludin expression was lowered in the duodenum during fructose feeding without changes in microbiota.	Increased intestinal translocation of microbial components is involved in the onset of fructose-induced NAFLD.	Wagnerberger et al. (52)
	high-fat diet plus 30% fructose solution (HFHF)	HFHF diet promoted changes in intestinal tight-junctions proteins, increased insulin resistance and plasma cholesterol. HFHF increased hepatic Lipocalin 2 (*Lcn2*) mRNA expression and plasma levels indicating hepatic inflammation.	Diets high in fat and fructose increase the vulnerability to metabolic syndrome-related conditions associated with NAFLD.	de Sousa Rodrigues et al. (7)
	30% fructose solution for 8 weeks with or without *Lactobacillus rhamnosus GG*	*Lactobacillus rhamnosus GG* increased number of beneficial bacteria, reduced duodenal IkB protein levels and restored the duodenal tight junction proteins. Portal LPS and hepatic expression of TNF-α, IL-8R and IL-1β was reduced and increase of fat accumulation and alanine-aminotransferase was attenuated.	Treatment with *Lactobacillus rhamnosus GG* protects against fructose-induced NAFLD.	Ritze et al. (53)
	30% fructose solution for 8 weeks and combination of bile acids chenodeoxycholic acid and cholic acid	The additional treatment with bile acids downregulated hepatic TNF-α, SREBP1, FAS mRNA expression, and lipid peroxidation. Bile acid treatment normalized expression of occludin, markers of Kupffer cell activation, and portal endotoxin levels.	Bile acids prevent fructose-induced hepatic steatosis through mechanisms that protect against the fructose-induced translocation of intestinal bacterial endotoxin	Volynets et al. (38)
	30% fructose solution for 8 weeks with or without concomitantly treatment with metformin (300 mg/kg body weight/day) in drinking water	Chronic consumption of fructose caused a significant increase in hepatic triglyceride and plasma AST levels. This effect was attenuated by metformin, which protected against loss of the tight-junction proteins occludin and zonula occludens-1 in the duodenum, thereby preventing increased translocation of bacterial endotoxin.	Metformin protects the liver from the onset of fructose-induced NAFLD through mechanisms involving its direct effects on hepatic insulin signaling and by altering intestinal permeability and subsequent endotoxin-dependent activation of Kupffer cells.	Spruss et al. (54)
	Sugar- and fat-rich Western-style diet (WSD) for 12 weeks plus fructose-supplemented water (30%)	Fructose intake increased endotoxin translocation, induced a loss of mucus thickness in the colon (246%) and reduced defensin expression in the ileum and colon. Microbiota analysis revealed that fructose increased the *Firmicutes*/*Bacteroidetes* ratio.	The consumption of a WSD or high fructose differentially affects gut permeability and microbiome. Fructose, especially when combined with a WSD, results in pronounced gut barrier dysfunction.	Volynets et al. (55)
	30% fructose solution, a high-fat diet, or a combination of both for 8 and 16 weeks	The combined diet induced development of hepatic steatosis and progression to steatohepatitis. Bacterial endotoxin levels in portal plasma increased, while levels of the tight junction protein occludin and zonula occludens-1 were reduced in the duodenum of all treated groups after 8 and 16 weeks.	Chronic intake of fructose and/or fat lead to the development of NAFLD over time which is associated with an increased translocation of bacterial endotoxin.	Sellmann et al. (56)
	Normal diet and high fat diet (HFD) with or without fructose for 16 weeks	Livers of mice fed with HFD and fructose showed a higher infiltration of lymphocytes and a lower inflammatory profile of Kupffer cells than livers of mice fed with the HFD without fructose. In the resulting dysbiosis, fructose specifically prevented the decrease of mouse intestinal bacteria in HFD-fed mice and increased *Erysipelotrichi*, independently of fat amount.	Fructose induces dysbiosis which is modulated by the presence of dietary fat. Combined diet of fat and fructose prevents fat-induced activation of Kupffer cells.	Ferrere et al. (57)
	High fat (40%)/high fructose (10%) diet with or without *Lactobacillus paracasei* supplementation for 10 weeks	Hepatic fat deposition, serum ALT level, and urinary ^51^Cr-EDTA clearance were significantly lower when mice received *L. paracasei*. The probiotics caused lower expression of TLR4 protein and mRNA for TNF-α, IL-4, MCP-1, PPAR-γ, and PPAR-α. The activation of Kupffer cells was lowered by *L. paracasei*.	*Lactobacillus paracasei* attenuates hepatic steatosis and Kupffer cell activation during progression of NASH.	Sohn et al. (48)
Rat	Diet enriched in fat and fructose	The diet induced a marked (i) increase in *A. muciniphila* in cecal microbiota, (ii) dramatic changes in the colon mucosa-associated microbiota, with a significant decrease in total bacteria, *Clostridium leptum, Bacteroides/Prevotella* and *Lactobacillus/Leuconostoc*, (iii) decreased expression of claudin-1, and (iv) increased expression of *Tnf-*α and *Tlr4*.	Diets enriched in fructose reduce bacterial colonization, lead to dysbiosis, increase numbers of mucin-degrading bacteria, and provoke inflammation in colon mucosa, thereby supporting NAFLD progression.	Jegatheesan et al. (58)
	Fructose-rich diet in combination with antibiotics for 8 weeks	After 4 weeks of treatment, fructose-fed rats exhibited higher values of fasting plasma insulin and homeostatic model assessment (HOMA) index. Antimicrobial therapy prevented diet-induced decrease of ileal occludin expression, increase of hepatic transaminases, lipid oxidation, and increase myeloperoxidase activity in ileum, liver, and visceral white adipose tissue. Similarly, quantities of portal TNF-α and LPS, as well as ileal TNF-α were induced by fructose. Fructose increased levels of plasma and hepatic triglycerides, irrespectively of antimicrobial treatment. Fructose increased oxidative damage to mitochondrial lipids and proteins, together with a significant decrease in antioxidant activity, while antibiotic treatment reversed all of these effects. A diet-dependent increase in *Coprococcus* and *Ruminococcus* was prevented by antibiotics.	Fructose promotes alterations in the gut microbiota profile triggering inflammation and metabolic dysregulation in the gut, liver, and visceral white adipose tissue. These obesity-related features can be experimentally reversed by treatment with antibiotics.	Crescenzo et al. (59)
	Diet enriched in copper combined with drinking water containing 30% fructose	The abundance of 38 fecal metabolites changed after dietary doses of copper or high fructose. Four SCFAs (valeric acid, butyric acid, isovaleric acid, and isobutyric acid) showed major abundance changes. The bacterial-derived long-chain fatty acid margaric was increased by excessive fructose intake.	Dietary fructose modifies the gut microbiota phylum profile contributing to the metabolic phenotype in NAFLD.	Wei et al. (60)
	60% isonitrogenous fructose diet for 4 weeks	Isonitrogenous fructose diet decreased *Bifidobacterium* and *Lactobacillus* and tended to increase endotoxemia without altering glucose homeostasis, liver function, or gut permeability.	Fructose provokes dysbiosis and fructose-induced hepatic alterations associated with NAFLD can be blunted by nitrogen supply.	Jegatheesan et al. (46)
	HFD for 5 weeks with or without a synbiotic composed out of *Lactobacillus fermentum* (CECT5716) and fructooligosaccharides	HFD for 5 weeks caused hepatic steatosis, insulin resistance, endotoxemia, increased production of SCFA, and increased numbers of *Bacteroidetes* in feces with an augmented *Bacteroidetes*/*Firmicutes* ratio. In addition, HFD weakened barrier function with increased LPS plasma levels. Saturation of absorptive mechanisms for fructose increased fructose availability in the distal and dysbiosis. Treatment with the synbiotic prevented some of the pathological effects, improved dysbiosis, and barrier function.	The synbiotic composed of *L. fermentum* CECT5716 and fructooligosaccharides has beneficial effects in the pathogenesis of HFD-induced metabolic syndrome.	Rivero-Gutiérrez et al. (61)
	70% (w/w) high-fructose diet for 3 weeks with or without oral addition of *Lactobacillus curvatus* and *Lactobacillus plantarum*	Fructose increased plasma glucose, insulin, triglycerides, total cholesterol, oxidative stress, liver mass, and liver lipids. Probiotic treatment lowered plasma glucose, insulin, triglycerides, and oxidative stress levels, while liver mass and cholesterol were only reduced at high-doses of probiotics. Probiotic treatment reduced lipogenesis *via* downregulation of SREBP1, FAS and SCD1 mRNA and increased β-oxidation *via* upregulation of PPARα and CPT2.	The combined administration of probiotic *L. curvatus HY7601* and *L. plantarum KY1032* suppress the clinical characteristics of high-fructose-induced metabolic syndrome.	Park et al. (37)
	Fructose-rich diet for 8 weeks combined with oral treatment with either antibiotics or fecal samples from control rats	The fructose-rich diet induced markers of metabolic syndrome, inflammation, oxidative stress and numbers of *Coprococcus* and *Ruminococcus*. These effects were reduced by both antimicrobial therapy and fecal treatments.	The development of fructose-induced metabolic syndrome is correlated with variations in the gut content of specific bacterial taxa.	Di Luccia et al. (62)
	10% fructose in drinking water for 6 weeks plus orally administered Juglanin	Juglanin prevented fructose-induced systemic increase of LPS levels, ALT, AST, ALP, and upregulation of TNF-α, IL-1β, IL-6, and IL-18. The flavonol suppressed fructose-feeding-induced activation of signaling pathways related to hepatic injury and inflammation.	Juglanin represses inflammatory responses and apoptosis through TLR4-regulated MAPK/NF-κB and JAK2/STAT3 signaling pathways.	Zhou et al. (63)
Monkey	Chronic *ad libitum* and short-term calorically controlled consumption of a high-fructose diet	Fructose increased biomarkers of liver damage, endotoxemia, and microbial translocation index.	Fructose rapidly causes liver damage secondary to changes in endotoxemia levels and microbial translocation.	Kavanagh et al. (64)
Human	Fructose feeding study	After 24-h fructose feeding, endotoxin levels in NAFLD adolescents increased after fructose beverages (consumed with meals) as compared to healthy children. Similarly, endotoxin was significantly increased after adolescents consumed fructose beverages for 2 weeks.	Fructose induces low level endotoxemia contributing to pediatric NAFLD.	Jin et al. (65)

In addition, the microbiota itself was shown to contribute to the progression of liver disease and injury and diet-induced NAFLD resulted in dysbiosis and a strong decrease in microbial diversity (66). Although the underlying mechanisms are not fully discovered yet, fructose is known to be highly involved in the development of NAFLD by altering gut metabolites (52, 60). Fructose increased the intestinal translocation of endotoxins and endotoxin levels in plasma (49, 64) contributing to inflammation and degrading of the mucosa barrier. Consequently, acute and chronic exposure to high fructose increased circulating endotoxin in patients with NAFLD, accompanied by markers of insulin resistance and inflammation (65). Interestingly, hepatic damage implicated by epitopic fat deposition occurred rapidly and significantly in non-human primates, even in the absence of weight gain (64) suggesting that arising periportal inflammation is the result of bacterial products overwhelming immune system and being presented to Kupffer cells (64). Altogether these studies indicate that fructose is able to induce inflammation promoting the development of NAFLD in two ways: it contributes to the formation of excessive fat triggering inflammation and it causes microbial dysbiosis promoting NAFLD pathogenesis.

In line, NAFLD and NASH patients were shown to consume more carbohydrates and particularly fructose (50). In a study, in which rats were fed a diet enriched in fructose, Wei et al. showed that the fecal metabolome profile was associated with the dietary fructose (60). Moreover, the chosen diet provoked formation of high quantities of SCFAs as well as C15:0 and C17:0 long chain fatty acids that are only produced by a special set of bacteria. This suggests that fructose-induced alterations in microbiota and not fructose itself are responsible for alterations in metabolome profile.

## Fructose Savages the Intestinal Barrier

**Figure 1:**
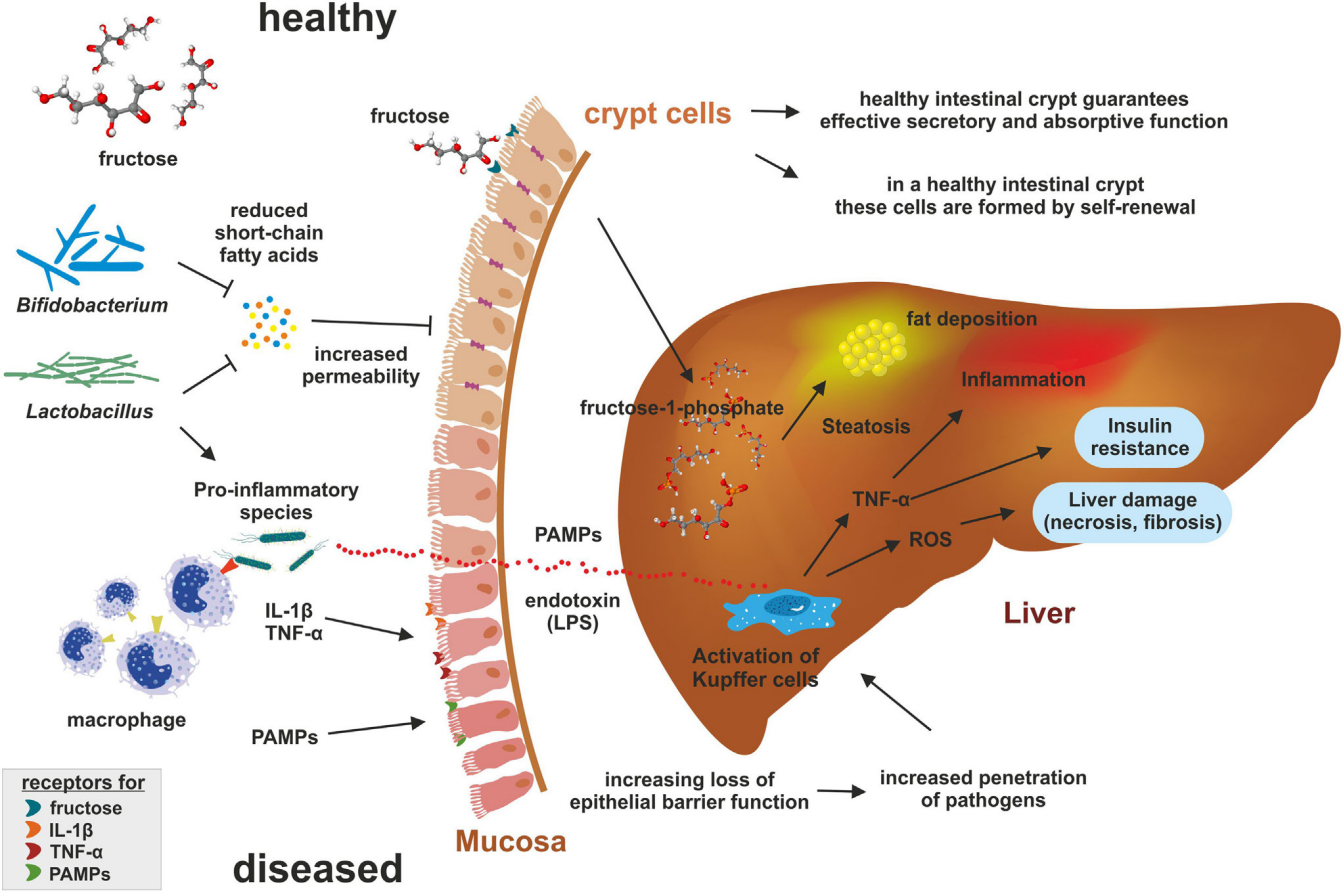
Impact of fructose on microbiome. Under healthy conditions, the intestine is organized into large numbers of self-renewing crypt-villus units guaranteeing effective secretory and absorptive functions. Elevated concentrations of fructose favor pro-inflammatory microbiota producing endotoxins and suppressing production of short-chain fatty acids (SCFA) that are essential for intestinal barrier function. Pro-inflammatory microbiota and their products [i.e., lipopolysaccharide (LPS); pathogen-associated molecular patterns (PAMPs)] recruit macrophages and bind to toll-like receptors (e.g., TLR4) leading to the release of cytokines such as tumor necrosis factor-α (TNF-α) causing mucosal inflammation. Subsequently, inflammation decreases expression of tight junction proteins resulting in a higher permeability of the gut barrier. In addition, endotoxins enter the leaky barrier leading to epithelial disruption and increase penetration of pathogens into the blood stream. Reaching the liver, endotoxins increase inflammation by activation Kupffer cells through binding to TLR4 and formation of reactive oxygen (ROS). The formed radicals induce hepatic damage and fibrosis. Furthermore, in the liver the fructokinase generate fructose-1-phosphate from fructose that is degraded into products providing substrate for *de novo lipogenesis* promoting steatosis.

The intestinal barrier represents a physiological boundary protecting the host by preventing entry of intestinal microbiota and microbial products (Figure 1). In particular, tight junctions, adherens junctions, and desmosomes connect epithelial cells, which together form a selective permeable epithelial barrier limiting the penetration of potentially pathogen substances (47).

Chronic intake of fructose is associated with a loss of tight junction proteins in the duodenum, elevated translocation of endotoxin, and induction of toll-like receptors (TLRs) in the liver (50, 53, 54, 56, 58). TLRs can be activated by microbial pathogen-associated molecular patterns (PAMPs). LPS is the most common PAMP, occurring in the cell membrane of gram-negative bacteria. LPS binds to its receptor TLR4, inducing nuclear translocation of transcription factor nuclear factor kappa (NF-κB)-light-chain enhancer of activated B cells resulting in an increased expression of pro-inflammatory cytokines including tumor necrosis factor-α (TNF-α), interleukin-6 (IL-6), and interleukin-1β (IL-1β) (63). Especially the tight junction proteins occludin and claudin-1 have been shown to decrease during fructose consumption (53, 58, 67). This process causes mucosal inflammation and intestinal epithelial barrier disruption, increasing the translocation of microbial products (47). A recent study of Volynets et al. pointed out that a sucrose-rich diet and a fructose-rich diet affected the intestinal microbiome in different ways (55). While a Western-diet high in sucrose primarily promoted weight gain, the intake of fructose, especially in combination with a Western diet, caused barrier dysfunction accompanied with loss of mucus thickness and endotoxin translocation (55).

Mice with a knockout in the *F11r* gene encoding the tight junction adhesion molecule A showed increased infiltration of intrahepatic macrophages, elevated recruitment of inflammatory monocytes to the liver, upregulation of TLRs and higher content of inflammatory cytokines when fed a diet high in fructose and fat (47). High fructose consumption promotes gut inflammation accompanied by rising endotoxin release, epithelial dysfunction, and decline of tight junctions proteins (50, 55, 56) independent of the fat content in the diet and energy intake (55). These data again illustrate the high impact of nutritional fructose on the intestinal barrier function.

The hepatic portal system connects the liver and the intestine, commonly called the “gut-liver-axis” (44). Consequently, the liver is the first organ exposed to gut-derived exotoxins, receiving 70% of the blood supply from the intestine. Therefore, the liver acts as a first defense against bacterial pathogens possibly explaining the relation between the influx of endotoxin and the progression of NAFLD and other liver diseases (68). Additionally, the gut microflora is able to stimulate hepatic fat deposition contributing to NAFLD and NASH (68). Fructose-induced impairment of intestinal gut barrier function enables the entering of bacterial products into the portal vein system and the liver, leading to Kupffer cell-mediated activation of inflammasomes and inflammation, increased formation of reactive oxygen species and proinflammatory cytokines such as TNF-α that are major causes of insulin resistance and dyslipidemia (16, 50, 67). Long periods of feeding a high fructose and high fat cause a rise in serum LPS, liver TLR4 expression and circulating cytokines suggesting that LPS and TLR4 are key molecules in the pathogenesis of NAFLD (3, 44, 47). In combination with dysbiosis, impaired gut function can promote metabolic endotoxemia as it was observed in animals that received a high sugar diet (3).

In addition, endotoxins are able to damage hepatocytes, causing activation of Kupffer cells that produce and release inflammatory cytokines and oxygen radicals. These products further aggravate liver damage (44). In line, mice fed fructose, fat or a combination of both showed elevated endotoxin and TLR4 levels, which were associated with changing the polarization of Kupffer cells and infiltrating macrophages, contributing to NAFLD (56). Infiltration of lymphocytes in the liver was increased when mice were fed a high fat diet supplemented with high fructose compared to mice fed a high fat diet alone (54, 69). In the respective model, fructose consumption induced increased lymphocyte recruitment that was accompanied by higher inflammation indicated by the elevated mRNA expression of TNF-α (54).

## Fructose Causes Dysbiosis

**Figure 2:**
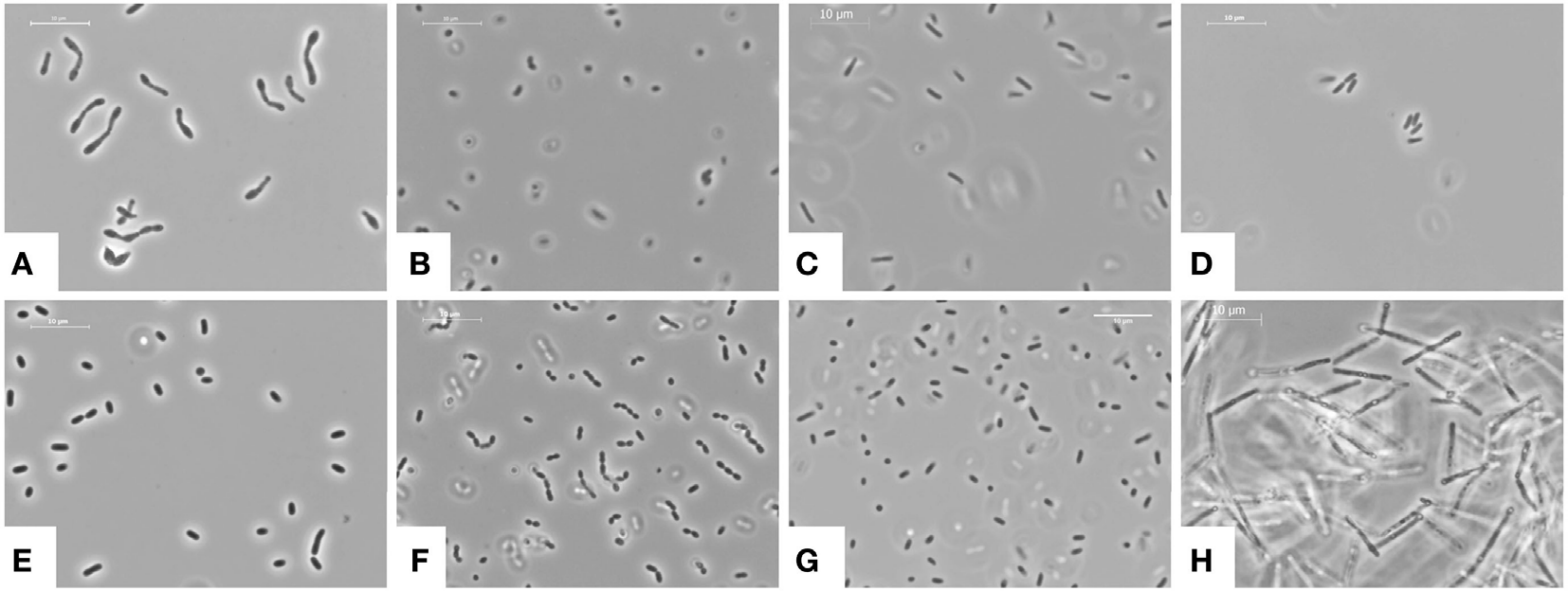
Bacterial phyla colonizing the healthy or diseased gut. **(A)** *Bifidobacterium longum* is a gram-positive, rod-shaped, health-promoting lactic acid bacterium present in the human gastrointestinal tract contributing to the production of butyrate. **(B)** *Bacteroides thetaiotaomicron* is a gram-negative, anaerobic microbe which dominates the intestinal tract flora of most mammals and provides the host with metabolic capabilities. **(C)** *Enterobacter cloacae* is a gram-negative, facultative-anaerobic, rod-shaped bacterium of the normal gut flora helping to debranch organic substances for energy production. **(D)** *Citrobacter freundii* is a common component of the gut microbiome of healthy humans. It is a facultative anerobic, rod-shaped gram-negative bacteria. **(E)** *Escherichia coli* is a gram-negative, facultative anaerobic, rod-shaped, “coliform” bacterium, which is commonly found in the lower intestine of warm-blooded organisms. Although most *E. coli* strains are harmless and part of the normal gut flora, some serotypes are occasionally responsible for food contamination causing serious intoxication in their hosts. They have capacity to produce vitamin K2 and prevent colonization of the intestine with pathogenic bacteria. **(F)** *Enterococcus faecalis* is a gram-positive, commensal bacterium inhabiting the gastrointestinal tracts. They are often arranged in pairs or in chain form and have both an anaerobic and aerobic metabolism. **(G)** *Salmonella enterica* is a gram-negative bacterium, flagellated, facultative anaerobic with a rod-shaped phenotype. A number of *Salmonella* variations are serious human pathogens provoking (spontaneous healing) diarrhea. **(H)** *Clostridium difficile* is a gram-positive, anaerobic, spore-forming bacterium able to produce multiple toxins causing diarrhea and inflammation. It may become opportunistically established in the human colon during antibiotic therapy. **(A–H)** All images were taken from cultures deposited in the national Culture Collection of Switzerland AG (CCOS, Wädenswil, Switzerland, https://www.ccos.ch/). The respective CCOS strain numbers are: CCOS 606, CCOS 632, CCOS 668, CCOS 669, CCOS 684, CCOS 688, CCOS 739, and CCOS 958. All images were taken using a phase contrast microscope at 1,000×. Space bars, 10 µm.

The structure and biology of gut microbiota are highly individually so that individuals can be identified simply on the basis of their “microbiota fingerprint” (15). Mostly six different bacterial phyla colonize the healthy gut (Figure 2), namely *Firmicutes, Bacteriotedes, Proteobacteria, Actinobacteria, Fusobacteria*, and *Verrucomicrobia* (70). About 90% of total bacteria in the gut of an adult are represented by three major divisions, the *Firmicutes* (gram-positive), *Bacteroidetes* (gram-negative), and *Actinobacteria* (gram-positive) (15).

Microbial diversity significantly decreases when consuming a high sugar diet already after 1 week, independent of the fat content (3). Fructose was shown to induce a different pattern of dysbiosis than a high fat diet.

Ferrere et al. found increased hepatic lymphocyte infiltration when mice were fed with a high fat and fructose diet compared to mice fed with high fat without fructose (57). Dysbiosis was observed in several studies when fructose was added to a normal diet (57). Mostly, a decrease of *Bacteriodes* and an increase of *Firmicutes* were observed when consuming a Western diet (27, 71–73). Independent studies comparing lean and diet-induced obese subjects suggested that *Firmicutes* promotes body fat accumulation (71, 72). Turnbaugh et al. reported that *Firmicutes* helps obese subjects to get more calories from the ingested food, which in turn leads to obesity (74). Also an increase in *Betaproteobacteria*, genera *Sutterella* was associated with enhanced hepatic fat deposition (3). Other studies demonstrated *Proteobacteria* as the main bacteria contributing to hepatic fibrosis and liver damage (47, 66). Furthermore, *Proteobacteria* is observed to be increased in several forms of dysbiosis and associated with NAFLD (75).

Rats subjected to different diets, marked changes in the microbiota already occurred already 1 week after the dietary switch to a diet enriched with high fat and high sugar (3). In a kind of circle, the increase in body fat mass correlated with shifts in the gut microbiota and gut-brain communication, possibly providing the basis for the pathogenesis of obesity (3).

Jegatheesan reported about alterations in colon mucosa-associated microbiota feeding a Western diet high in fat and fructose for 8 weeks (58). This was associated with lower levels of *Clostridium leptum, Bacteroides/Prevotella*, and *Lactobacillus/Leuconostoc*, while the content of *Akkermansia muciniphila* was not affected (58). *A. muciniphila* is known to strengthen the epithelial barrier function and to impact the systemic health of the host (76). It has been shown that this cross-talk between the host and gut microbiota protects against high fat diet-induced LPS endotoxemia in obese mice (77). In the above mentioned study of Sen et al., a high fat/high sugar diet resulted in a specific increase in *Anaeroplasmatales*, which are an order of *Mollicutes* bacteria, a class of *Tenericutes* which is linked to diet-induced fat deposition and obesity (3). Adipose tissue inflammation plays a major role in the pathogenesis of NAFLD, intestinal dysbiosis might therefore be an additional factor leading to disease progression (78).

In line with this assumption, Raman et al. discovered a significant difference in the fecal microbiome in NAFLD patients and healthy subjects (75). In patients with NAFLD, the dysbiosis is mainly characterized by a decrease in *Bacterioides* (*Prevotella*) and an increase of *Clostridium coccoides* (45). Consistently, patients with IBS showed a similar dysbiosis characterized by an increase in *Clostridium* cluster XIVa and *Ruminococcaceae* with a concomitant decrease in *Bacteroidetes* and *Bifidobacteria* (79). In a more recent study, Xue et al. detected an increase of serum LPS and aerobic bacteria, such as *Escherichia coli* and *Enterococcus* and a decrease in the amounts of *Lactobacillus, Bifidobacteria*, and *Bacteriodes* in rats that were subjected to diets inducing experimental NAFLD (44).

De Minics et al. investigated the transformation of the microbiome in animal models of NAFLD, and compared high fat diet-induced NAFLD and bile duct ligation-induced liver damage. In the respective study, it was found that intestinal permeability and bacterial translocation are the key pathogenetic events triggering progression of liver damage. Moreover, the authors showed that the main effector in the degree of liver damage is related to microbiota changes (66). Similarly, fructose induced alterations in the microbiome of rats were associated with metabolic dysregulation and inflammation in gut, liver, and fat tissue that could be attenuated by antibiotic treatment or treatment with control fecal samples (59, 62). These observations again underpin the importance of microbiota in the development of metabolic diseases. Therefore, the authors of respective studies concluded that manipulation of the gut bacteria interferes with liver injury and progression of NAFLD.

To sum up, the human gut is colonized by several strains of microbiota, some with pro- and some with anti-inflammatory effects. A healthy gut is characterized by a homeostasis requiring a balance between the gut flora and the immune system of its host. Triggers that provoke intestinal leakage such as fructose cause a shift in this homeostasis favoring proinflammatory microbiota, suppression of anti-inflammatory microbiota, and reduction of their overall diversity.

## Fructose-Induced Production of Uric Acid Further Provokes Liver Damage

**Figure 3:**
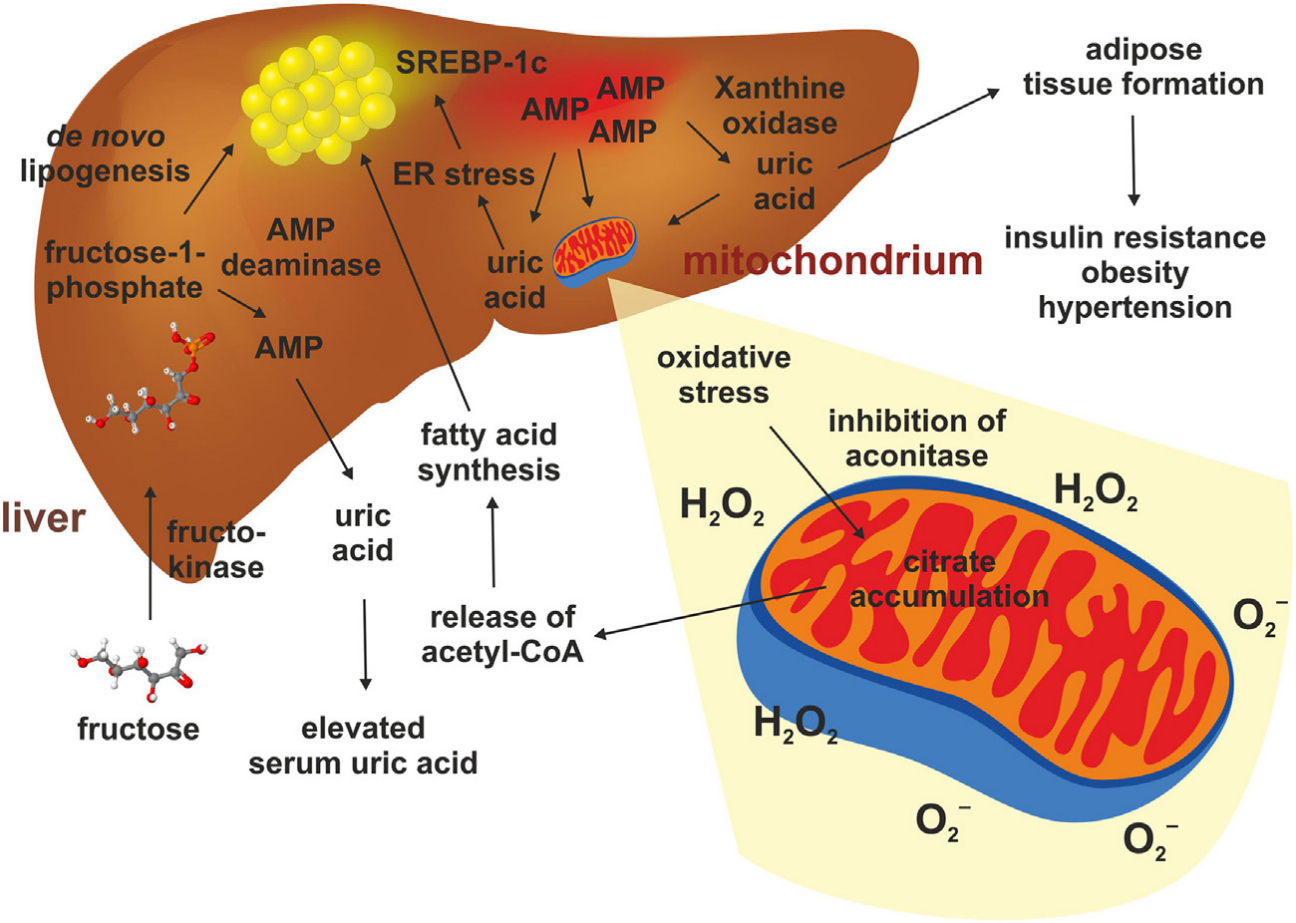
Hepatic fructose metabolism and uric acid. The metabolism of fructose in the liver starts with a phosphorylation to fructose-1-phosphate. This happens quickly and causes an accumulation of AMP, which stimulates the AMP deaminase and the xanthine oxidase. This results in elevated production of uric acid in liver and serum. Uric acid triggers adipose tissue formation, glucose intolerance, elevated blood pressure, and dyslipidemia. Uric acid also promotes mitochondrial oxidative stress and release of superoxide anion $\left({\text{O}}_{2}^{-}\right)$ and hydrogen peroxidase (H_2_O_2_). This gives rise to reduced aconitase activity resulting in citrate accumulation. This results in increased cytosolic acetyl-CoA which is a substrate for *de novo* lipogenesis. In addition, uric acid can induce endoplasmatic reticulum stress in hepatocytes, thereby leading to a direct activation of genes such as SREBP-1c stimulating hepatic steatosis.

It was demonstrated that a Western diet leads to an increase in uric acid within the blood and that measured serum levels of uric acid directly correlate to the intake of fructose (80). The primary producers of uric acid are the hepatocytes and an anomalous metabolism of uric acid causes hepatocyte damage and produce oxidative stress (81). When fructose from dietary sources is absorbed through the fructose transporter GLUT5 within the intestinal epithelium and transported to the liver, it is rapidly phosphorylated in the liver by fructokinase, causing hepatic accumulation of fructose-1-phosphate (F-1-P) and a simultaneously increase in AMP (82) (Figure 3). Subsequently, the elevated hepatic concentration of F-1-P induces changes of several other metabolites such as glucose, lactate and uric acid (82). F-1-P is converted in triosephosphate providing substrates for *de novo* lipogenesis (DNL) (80, 82). Simultaneously, the decrease in intracellular phosphate stimulates AMP deaminase stimulating the generation of uric acid *via* xanthine oxidase and production of superoxide anion $\left({\text{O}}_{2}^{-}\right)$ and hydrogen peroxidase (H_2_O_2_) (80, 83, 84). This process is reinforced under inflammatory conditions (80). Lanaspa et al. suggested that uric acid plays a pivotal role in the lipogenic ability of fructose. In the respective study, triglyceride accumulation was decreased by adding an inhibitor of xanthine oxidase (84). Likewise, Choi et al. showed that uric acid induced fat accumulation in HepG2 and in primary hepatocytes as a result of endoplasmatic reticulum stress induction, which could activate SREBP-1c and stimulate steatosis (85).

Mitochondrial oxidative stress inhibited aconitase resulting in accumulation of cytosolic citrate. This tricarboxylic acid promoted DNL by activating ATP-sensitive lipase converting citrate to acetyl-CoA, thereby inducing fatty acid synthesis (84). Simultaneously, oxidative stress in the liver mitochondria induced by uric acid leads to alterations of mitochondrial function and cell damage (81, 84). Even a single administration of fructose attenuates uric acid excretion in the ileum (83), and long-term consumption of fructose was shown to suppress renal uric excretion resulting in increased serum uric acid levels (86). Consequently, high uric acid impaired glucose tolerance, causing insulin resistance and inhibition of insulin signaling (87).

## Improvement of Diet-Induced NAFLD

**Figure 4:**
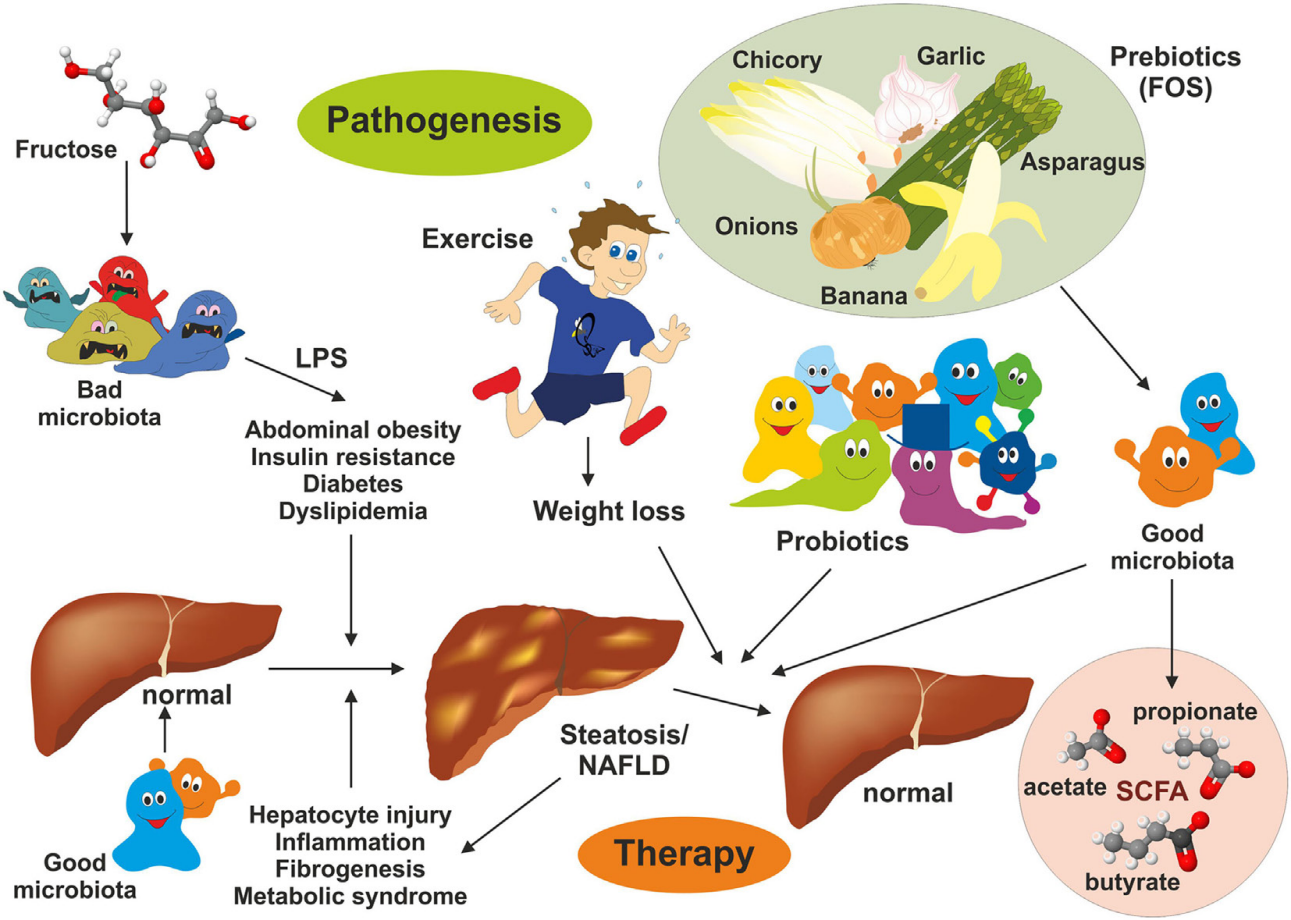
Intervention strategies for the treatment of non-alcoholic fatty liver disease (NAFLD). NAFLD is a multifactorial disease manifesting in liver steatosis, hepatocyte injury, inflammation, and fibrogenesis. It is further associated with abdominal obesity, insulin resistance, diabetes, and dyslipidemia. Recent evidence suggests that the gut microbiome represent a significant environmental factor contributing to NAFLD. Dysbiosis induces deregulation of the gut endothelial barrier function facilitating bacterial translocation. Bacterial-derived products [e.g., lipopolysaccharide (LPS)] are key driver of hepatic inflammation. Although effective pharmacological therapies for NAFLD are not available, lifestyle changes (modulation of diet), exercise, and weight loss have been shown to be beneficial on NAFLD outcome. Supplementation with probiotics and prebiotics restoring the microbial balance and changing the “*bad microbiota*” to “*good microbiota*” have health-promoting effects by generation of short-chain fatty acids (e.g., acetate, propionate, and butyrate) interfering with NAFLD progression.

Currently, there is no real effective drug therapy for treatment of NAFLD. However, interventions in lifestyles, health-promoting diets, application of probiotics, nutritional supplementation with prebiotics inducing growth or activity of beneficial microorganisms, and exercise resulting in weight loss (88) have been shown to improve NAFLD (Figure 4). Some aspects of the beneficial effects of each intervention are discussed later.

## Health-Promoting Effects of Mediterranean Diets

The traditional MD is based on high intake in mono- and polyunsaturated fatty acids derived from olive oil or fish, vegetables, fruits, and nuts providing high fiber entry. MD has been shown to correlate negatively with NAFLD, and in combination with salt restriction, MD was shown to lower blood pressure, improve blood lipids, and improve steatosis and steatohepatitis, while omega-3 polyunsaturated fatty acids have been shown to reduce accumulation of lipid and liver enzymes, improve insulin sensitivity, and act as an anti-inflammatory compound (8). The high amount of fibers in the MD is accompanied by polyphenols, antioxidants and phytochemicals, plant metabolites capable to inhibit DNL, liver steatosis, and inflammation. Furthermore, fiber and phytochemicals enriched in whole grain are able to reduce energy intake, promote SCFA-producing gut bacteria, and have significant prebiotic effects. Based on these positive health-promoting effects, it is obvious that changing to a MD is one therapeutically effective mean to improve the outcome or severity of NAFLD (8).

## SCFA Influence Fat Metabolism *via* Activation of Adenosine Monophosphate Kinase (AMPK)

**Figure 5:**
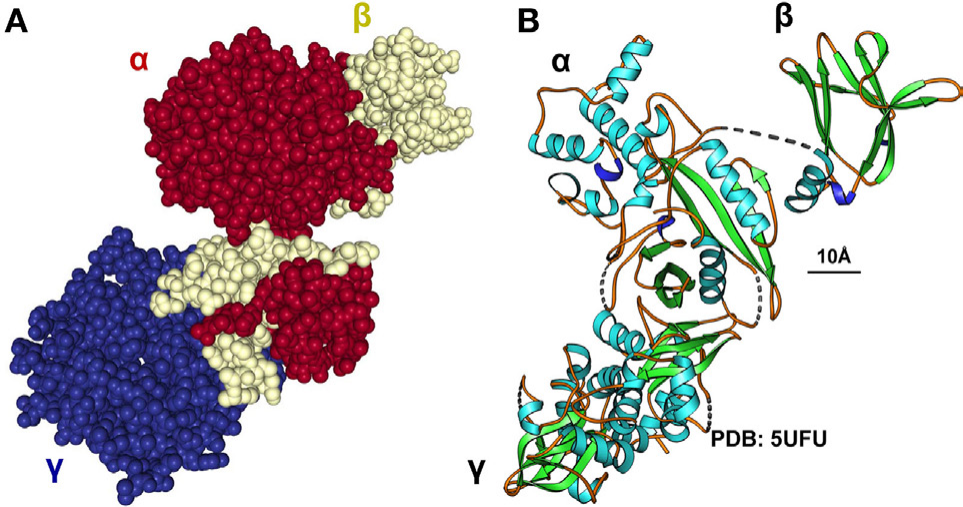
Structure, regulation and functional aspects of adenosine monophosphate kinase (AMPK) biology. **(A,B)** The AMPK is a heterotrimeric protein kinase complex comprised of α-, β-, and γ-subunits. AMPK is activated by phosphorylation of a critical threonine residue located within the α-subunit that is triggered by binding of AMP and/or ADP to the γ-subunit. ATP competitively inhibits the binding of both AMP and ADP to the γ-subunit suggesting that AMPK is a critical sensor of AMP/ATP or ADP/ATP ratios (93). The space bar represents 10 Å. The CPK representation in **(A)** was generated with the interactive web-based tool NGL (94) and the ribbon drawing in **(B)** with Ribbons XP Version 3.0 (95) using the structure coordinates 5UFU deposited in the PDB Brookhaven database. More structural details of human AMPK are given elsewhere (96, 97).

**Figure 6:**
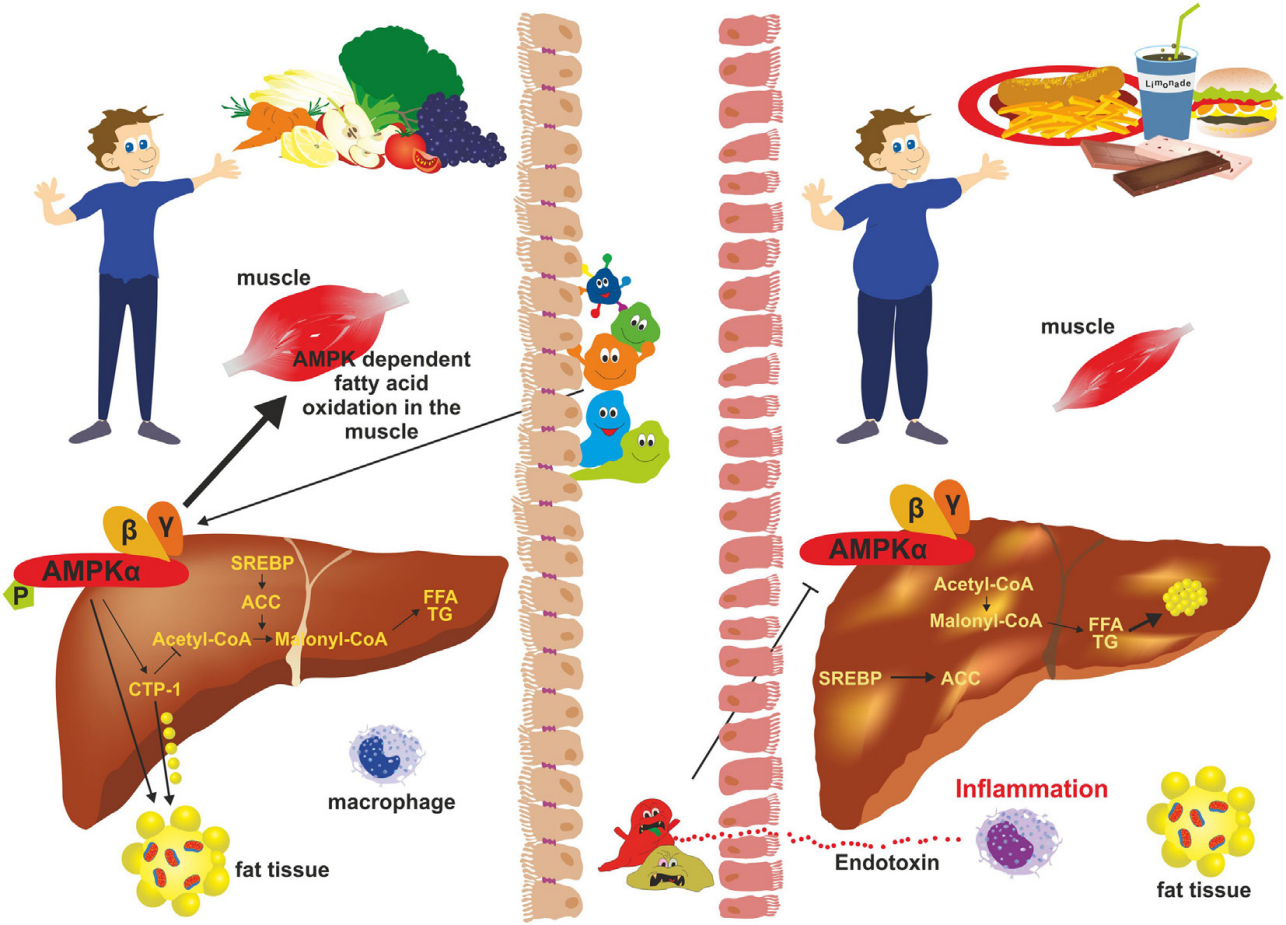
Adenosine monophosphate kinase (AMPK) activity and gut microbiota. In subjects with a balanced “good” microbiota (*left*), AMPK phosphorylates key regulatory factors [e.g., acetyl-CoA carboxylase 1 (ACC1); sterol regulatory element-binding protein 1c (SREB1c)] that inhibit synthesis of fatty acids, cholesterol, and triglycerides. It further stimulates β-oxidation and glucose uptake in skeletal muscle and inhibits gluconeogenesis in the liver. During dysbiosis (*right*), “bad” gut microbiota inhibits phosphorylation of AMPK thereby negatively influencing hepatic fatty oxidation and favoring lipogenesis resulting in excessive fat storage in the liver and obesity.

Adenosine monophosphate kinase is an important enzyme expressed mainly in the liver and skeletal muscles, playing a crucial role in cellular energy homeostasis (15). It is one of the central regulators of the body’s metabolisms by promoting catabolic pathways to generate more ATP and inhibition of anabolic pathway. The enzyme is a heterotrimer composed of a catalytic α-subunit and two regulatory subunits (β and γ) (Figure 5). The activity of AMPK is strongly influenced by the gut microbiota (Figure 6). Drugs increasing expression of AMPK stimulate fatty acid oxidation in liver and muscle tissues, resulting in energy loss and prohibition of obesity (89). Specifically, certain food compounds and dietary strategies are suitable to activate AMPK and to mediate antidiabetic effects. Some intestinal bacteria are able to digest polyphenols and isoflavones from plants and convert it to enterolactone (ENL) or to equol, which both activates AMPK (63, 90). Furthermore, ENL ameliorates abnormal lipid metabolism, inhibits anabolic glycogen storage, and improves insulin sensitivity (63, 90). In the respective study it was shown that ENL dose dependently promoted glucose uptake under insulin absent condition, which was completely eliminated by an AMPK inhibitor suggesting that ENL is an antidiabetic substance. Conversely, the inhibition of AMPK prevented fatty acid oxidation in several organs and tissues, promoted the synthesis of cholesterol and triglycerides, and favored lipogenesis leading to excess fat storage and obesity (28). Therefore, food enriched in such pharmacological active substances including grapes, plums, strawberries, passion fruit, white tea, and soy are predicted to improve diet-induced NAFLD. Another study focusing on the beneficial impact of butyrate on the gut barrier reported that butyrate activates AMPK and induced a redistribution of the tight junction proteins zonula occudens-1 (ZO-1) and occludin (91). Additionally, butyrate activated AMPK induced SIRT1 phosphorylation that influences glucose homeostasis and insulin sensitivity in a positive way (92).

## Probiotics

There is now ample evidence showing the importance of probiotics in host health. As already discussed above, the main bacterial bioproduct, which is associated with the pathogenesis of NAFLD, is LPS. Xue et al. detected a positive correlation between serum LPS and aerobic bacteria, such as *Escherichia coli* and *Enterococcus* and a negative correlation between the amount of *Lactobacillus, Bifidobacteria*, and *Bacteriodes* in rats subjected to NAFLD inducing diets (44). Interestingly, it was demonstrated in the respective study that the amounts of anaerobic bacteria such as *Lactobaiullus* and *Bifidobacteria* could be restored by probiotics (44). The application of probiotics ameliorated the intestinal barrier in NAFLD and tight junctions were shown to be more complete in comparison to NAFLD rats without probiotics (44, 48). Furthermore, serum levels of LPS and TLR4 decreased significantly after administration of probiotics in a NAFLD rat model. In addition, the livers of the respective animals showed less hepatocyte swelling, less cell infiltration, lower degree of inflammation, and overall milder steatosis (44, 48). Along with that, serum levels of triglycerides, total cholesterol, LDL, and free fatty acids were diminished. Interestingly, the amount of high density lipoprotein (HDL) was increased compared to the control group and animals exhibited better glucose tolerance in comparison to NAFLD rats without probiotic treatment (44). Moreover, different *Lactobacillus* strains were identified that diminished hepatic fat accumulation, increased of serum alanine aminotransferase (ALT) levels, and improved intestinal gut barrier in a NASH model (48, 51). The combination of multiple *Lactobacillus* strains reduced plasma glucose and insulin levels, triglycerides, and oxidative stress. Moreover, high probiotic treatment reduced liver mass and liver cholesterol by increasing β-oxidation and lowering the expression of sterol regulatory element-binding transcription factor 1 (SREBP-1), stearoyl-CoA desaturase-1 (SCD-1), and fatty acid synthase (FAS) mRNA levels that are critically associated with fructose-induced metabolic syndrome (37). In similar studies, *Lactobacillus casei* was shown to decrease fat storage, oxidative stress, and hepatic inflammation in a NASH mouse model and to diminish the activation of the TLR4 signaling cascade (51, 98).

In a rat model, treatment with the butyrate-producing gram positive strain *Clostridium butyricum* prevented the progression of nutrient-induced NAFLD (92). When given as dietary supplement (i.e., MIYAIRI 588), this bacterial strain suppressed the diet-induced increase in endotoxin levels, and restored the expression of the tight junction proteins ZO-1 and occludin (92). It altered the intestinal flora and restored gut-barrier functions, reduced hepatic levels of cytokines as TNF-α and was able to regulate the activation of NF-κB (92).

Toll-like receptor 4 is the main receptor that detects gut-derived endotoxins and regulates hepatic inflammation in NASH and probiotics were shown to decelerate the progress of NAFLD by inhibiting the LPS-TLR4-signaling pathway, improving intestinal flora dysbiosis, restoring normal gut homeostasis, and upregulating expression of tight junction proteins strengthening the gut barrier (44, 48). Patients with IBS exhibited a high activity of intestinal serine proteases, which could be biologically sequestered by *Bifidobacteria* acting as an antagonist of this endopeptidase (79). Therefore, probiotics are presently discussed as new therapeutics in clinical management of NAFLD (99).

## Prebiotics

**Figure 7:**
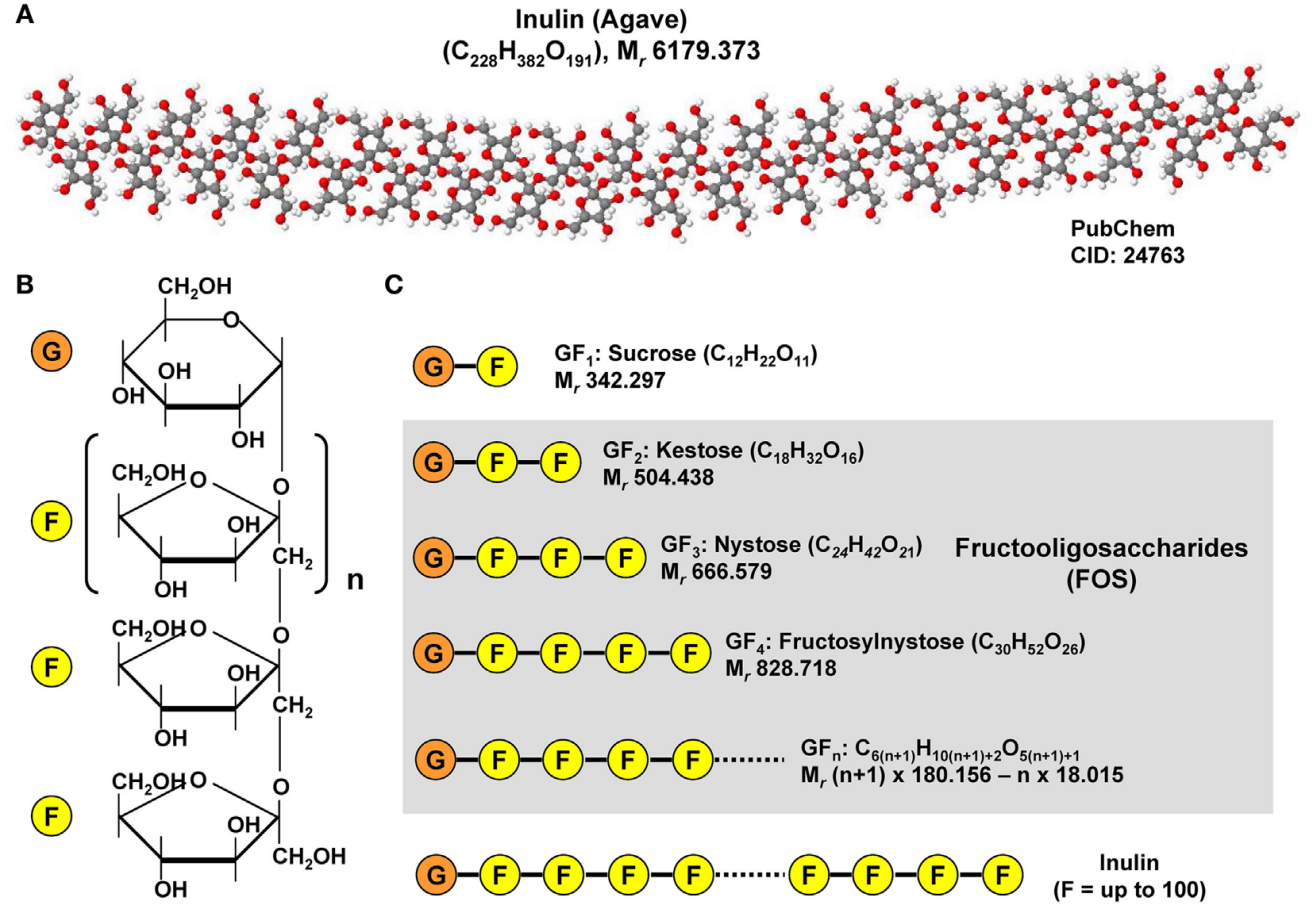
Chemical structure of inulin and fructooligosaccharides. **(A)** Inulins are naturally occurring indigestible polysaccharides belonging to the class of dietary fibers. Inulin from the blue Agave (*Agave tequilana*) is composed of linear and branched fructose chains that are connected *via* β-2,1 and β-2,6 linkages to each other with a total degree of polymerization between 25 and 34. The depicted structure was constituted with Jmol (version 14.2.15_2015.07.09) using the PubChem CID file 24763 deposited in the PubChem Compound Database. **(B)** Fructoligosaccharides contain a variable number of β-D-fructofuranosyl units with one glucosyl unit. **(C)** Representative fructooligosaccharides (FOS) are kestose (GF_2_), nystose (GF_3_), fructosylnystose (GF_4_) differing in number of fructose residues. The general molecular formula of a FOS is C_6(_*_n_*_+1)_H_10(_*_n_*_+1)+2_O_5(_*_n_*_+1)+1_ giving rise to a molecular mass of (*n* + 1) × 180.156 − *n* × 18.015 g/mol when *n* is the total number fructose residues.

In addition, gut microbial modulation can also be achieved by intake of substances that induce the growth or activity of beneficial microorganisms. These prebiotics act as substrate for respective bacteria and can be supplied as a functional food component. Prototypically, the plant polyphenol Quercetin found in many fruits has been shown to modulate diet-induced dysbiosis in mice with NAFLD by increasing the production of SCFA, thereby improving the intestinal gut barrier and inhibition of diet-induced hepatic inflammasome activation (100). In addition, Quercetin supplementation counteracted the upregulation of lipogenic genes and reduced the amount of *Helicobacter*, which is more frequent in diet-induced NAFLD (100). In a similar study from Baldwin et al., the feeding of powdered grape extracts reduced fat gain and hepatic lipid accumulation in a high fat mouse model (101). Also fructooligosaccharides (FOS) were shown to act as prebiotics in a metabolic syndrome model (61). FOS are composed of relative short chains of less than 20 molecules of fructose that are linked to a glucose terminal residue and cannot be digested by humans (Figure 7). They can be extracted from many plants (e.g., blue Agarve) and naturally occur in high concentration in many fruits and vegetables such as bananas, artichokes, onions, chicory root, garlic, asparagus, and leeks. In combination with *Lactobacillus fermentum*, FOS were effective in preventing intestinal permeability, systemic inflammation, hepatic steatosis and insulin resistance without inducing an innate immune system reaction at the intestinal level during high fat diet (61). Similarly, the mentioned study of Baldwin et al. showed that FOS mediated an increase in *A. muciniphila*, which was accompanied by decreased metabolic endotoxemia and reduced expression of inflammatory markers in white adipose tissue (101). Increased abundance of this mucin degrader residing in the mucus layer correlated with improvement of high-fat diet-induced metabolic disorders, reduction of obesity, reduced metabolic endotoxemia, adipose tissue inflammation, and insulin resistance (77).

## Physical Activity

It is already well established that exercise is associated with the production and release of potent, pharmacologically active, anti-inflammatory mediators, which can principally counteract liver inflammation and chronic low-grade inflammation (102). In a recent study, Batacan et al. investigated the combined effect of exercise and diet treatments on intestinal microbiota in a rat model (103). The authors found that the development of the microbiota in response to physical activity depends on basic starting microbiota. Moreover, training could induce a microbiota composition which is able to break down carbohydrates more effectively, improving the performance through energy provision, and increasing the production of SCFAs. Therefore, the authors suggested exercise as a factor for remodeling microbiota and gut health which is negatively influenced by high fat diets preventing microbiota differentiation in response to exercise (103).

## Conclusion

Non-alcoholic fatty liver disease is a systemic disease induced and modulated by different metabolic components, in which the liver is the main affected organ. Fructose consumption causes dysbiosis in the microbiota, leading to an increased permeability of the gut barrier, hepatic inflammation, progressive development of metabolic syndrome, and insulin resistance (57, 62, 69). Microbiota is highly influenced by diet and lifestyle of its host and a major factor influencing the outcome of metabolic diseases. Changing gut flora by intake of dietary fiber (i.e., roughage), probiotics, prebiotics, as well as intensifying frequency and duration of physical activity, leads to improvement of hepatic inflammation and fibrosis. These dietary and lifestyle interventions affecting microbiota are presently the most effective treatment options to improve NAFLD. Therefore, these nutritional and behavioral therapies should be combined with diets that lack excessive NAFLD-promoting compounds such as fructose and fat that are majorly contributing to the pathogenesis of NAFLD.

## Author Contributions

JL and RW have written this review. SW prepared the final Figures 1, 3, 4 and 6. SL provided images of bacterial cultures depicted in Figure 2. RW prepared Figures 5 and 7 and has made the final editorial assignments. All authors agreed to submit this review and to be accountable for the content of the work.

## Conflict of Interest Statement

The authors declare that the research was conducted in the absence of any commercial or financial relationships that could be construed as a potential conflict of interest.

## Abbreviations

AMPK, adenosine monophosphate kinase; BDL, bile duct ligation; DNL, *de novo* lipogenesis; ENL, enterolactone; F-1-P, fructose-1-phosphate; FOS, fructooligosaccharides; IBS, irritable bowel syndrome; IL-1β, interleukin-1β; IL-6, interleukin-6; LPS, lipopolysaccharide(s); MD, Mediterranean diet; NAFLD, non-alcoholic fatty liver disease; NASH, non-alcoholic steatohepatitis; PAMP(s), pathogen-associated molecule(s); SCFA, short-chain fatty acid(s); TLR(s), toll-like receptor(s); TNF-α, tumor necrosis factor α.
